# Significance of dynamically monitoring serum estrogen and β‐human chorionic gonadotropin in early pregnancy assessment

**DOI:** 10.1002/jcla.23559

**Published:** 2020-09-06

**Authors:** Yang Li, Jiaou Zhang, Kemei Zhang, Ensheng Wang, Jing Shu

**Affiliations:** ^1^ Reproductive Medicine Center Ningbo First Hospital Ningbo China

**Keywords:** dynamic monitor, estrogen, pregnancy assessment, β‐human chorionic gonadotropin

## Abstract

**Background:**

Spontaneous abortion occurs in 15% ~ 25% of clinical pregnancy. β‐human chorionic gonadotropin (β‐HCG) and progesterone (P) have been widely used in early pregnancy assessment, but their clinical significances are still controversial. Estradiol (E2) has not been used as widely as β‐HCG and P, and its value in predicting pregnancy outcome is unclear.

**Methods:**

In this retrospective study, two hundred early pregnancy women were divided into two groups according to their early pregnancy outcomes: the ongoing pregnancy group and inevitable abortion group. Serum E2 and β‐HCG levels and their growth rates were compared weekly.

**Results:**

Estradiol and β‐HCG of the ongoing pregnancy group were significantly higher than that of the inevitable abortion group from the 5th to 10th week of pregnancy. Taking 489.5 pg/mL in the 5th and 6th week, 590.5 pg/mL in the 7th week, and 614.5 pg/mL in the 8th week as cutoff levels of E2, the sensitivity and specificity for E2 to predict bad pregnancy outcome were 91.7% and 41.5%, 82.9% and 71.1%, 84.8% and 84.7%, 75.0% and 95.7%, respectively (*P* < .05). Both E2 and β‐HCG increased much more rapidly in the ongoing pregnancy group. 80% of the normal pregnancy women showed continuously increasing E2 level. Meanwhile, the inevitable abortion group presented E2 variation types as slow increase or fluctuation, continuous decline, and sudden drop, which account for 54.0%, 34.0%, and 12.0%, respectively.

**Conclusion:**

Low values and low growth rates of E2 and β‐HCG probably indicate bad pregnancy outcomes.

## Graphical abstract

Clinical significance of dynamic monitoring of E2 and β‐HCG for early pregnancy assessment and pregnancy outcome prediction were evaluated. Both E2 and β‐HCG increased much more rapidly in ongoing pregnancy women. Low values and low growth rates of E2 and β‐HCG probably indicate bad pregnancy outcomes.

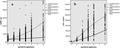

## 1 INTRODUCTION

Spontaneous abortion occurs in 15% ~ 25% of clinical pregnancy, among which about 5% women suffer from recurrent pregnancy loss.^1^, ^2^ Patients who have previous pregnancy loss experience tend to be more nervous and anxious, and are likely to take examination more frequently. In early pregnancy inspection, doctors hope to find cost‐effective indicators to evaluate the pregnancy conditions and predict pregnancy outcomes, so as to timely adjust the treatment strategy and accurately make the decision to provide active treatment or not. Serum β‐human chorionic gonadotropin (β‐HCG) and progesterone (P) have been widely used in early pregnancy assessment, but the clinical significance and value of progesterone test are still controversial. Another reproductive hormone estradiol (E2) is also very important in pregnancy maintenance, but has not been used as widely as β‐HCG and P. In this study, serum E2 and β‐HCG levels were analyzed in early pregnancy women, to explore the clinical significance of dynamically monitoring E2 and β‐HCG in assessing pregnancy conditions and predicting pregnancy outcomes.

## 2 MATERIALS AND METHODS

### 2.1 Patients

In this retrospective study, participants were early pregnancy women who visited the clinic between July 2017 and December 2018 in Ningbo First Hospital, China. We estimated the gestational age according to the date of last menstrual period of women with regular menstrual cycle. For women with irregular menstrual cycle, we determined the ovulation date by follicle monitoring through transvaginal ultrasound. Those who did not take follicle monitoring and could not provide the exact ovulation date were excluded. The inclusion criteria were intrauterine singleton pregnancy, and gestational age between 4 weeks and 10 weeks. The exclusion criteria were chemical pregnancy, ectopic pregnancy, multiple pregnancy, pregnancy through in vitro fertilization, chromosome abnormity in either of the couple, and treatment with estrogen or pills containing estrogen. Clinical information of the patients was recorded at their first visit. The study was approved by the ethics committee of Ningbo First Hospital.

### 2.2 Study design

Patients were divided into the ongoing pregnancy group and the inevitable abortion group according to their early pregnancy outcomes. The ultrasound scan to assess the fetal viability was performed using a 5 ~ 9 MHz transvaginal probe (GE Voluson E8; GE Healthcare Austria GmbH & Co OG) for women before 7 weeks of gestational age or a 3.5 ~ 5 MHz abdominal probe for women after 7 weeks of gestational age. Criteria of the ongoing pregnancy group included intrauterine pregnancy with primitive cardiac beat, and embryo size on ultrasonography corresponded to the calculated gestational age, without or just a little vaginal bleeding. Participants were classified to the inevitable abortion group when meeting any of the following conditions: persistent vaginal bleeding or abdominal pain, followed by expulsion of the embryo; failed to detect embryo's cardiac beat for two or more ultrasonic tests after 7 weeks of gestational age; and normal cardiac beat disappeared. The end of follow‐up time of this study was 12th week of gestational age.

Patients took venous blood test detecting serum reproductive hormones every one or two weeks until the 10th week of pregnancy. The serum E2 level was measured using the Access Estradiol kit (Beckman Coulter Inc.), with a sensitivity of 20 pg/mL. The coefficient of variation (CV) was ≤12% when E2 > 120 pg/mL, and CV was estimated to be 13% ~ 21% when E2 < 120 pg/mL. The serum β‐HCG level was measured using the Access Total β‐HCG kit (Beckman Coulter Inc.), with a sensitivity of 0.6 mIU/mL and CV ≤ 10% when β‐HCG > 3.9 mIU/mL. The E2 and β‐HCG levels were recorded and compared at 4 weeks ± 2 days, 5 weeks ± 2 days, 6 weeks ± 2 days, 7 weeks ± 2 days, 8 weeks ± 2 days, and 10 weeks ± 2 days of gestational age. Growth rates of the two hormones were analyzed and compared. Receiver operating characteristic (ROC) curves analysis of E2 from the 5th to 8th week of gestational age were generated.

We classified the variation of E2 into four types: a: persistent increase; b: slow increase or fluctuation; c: continuous decrease; and d: sudden drop. The four types of variation were compared between the two groups.

### 2.3 Statistical analysis

Statistical analysis of the clinical data was performed by SPSS Statistics version 19.0 (SPSS Inc., Chicago, IL, USA). Continuous data were compared with Student's *t* test or Mann‐Whitney *U* test for non‐parametric variables. Categorical data were compared with chi‐square test or Fisher's exact test where appropriate. ROC curves depicting predicted probabilities were generated from logistic regression models of pregnant outcomes. *P*‐values that are smaller than .05 were considered statistically significant.

## 3 RESULTS

Two hundred women were recruited in the study. The average age was 30.01 ± 3.88 years old, ranging from 23 to 42 years old. The ongoing pregnancy group included 150 women, aging at 29.73 ± 3.65 years old on average. 42 women (28%) had a history of recurrent pregnancy loss (two or more pregnancy losses, either clinical or chemical). 50 women suffered from inevitable abortion, aging at 30.82 ± 4.44 years old on average, among which 12 women (24%) had a history of recurrent pregnancy loss. There were no statistically significant differences between the two groups in terms of age (*P* = .123) and history of recurrent pregnancy loss (*P* = .581).

**Figure 1:**
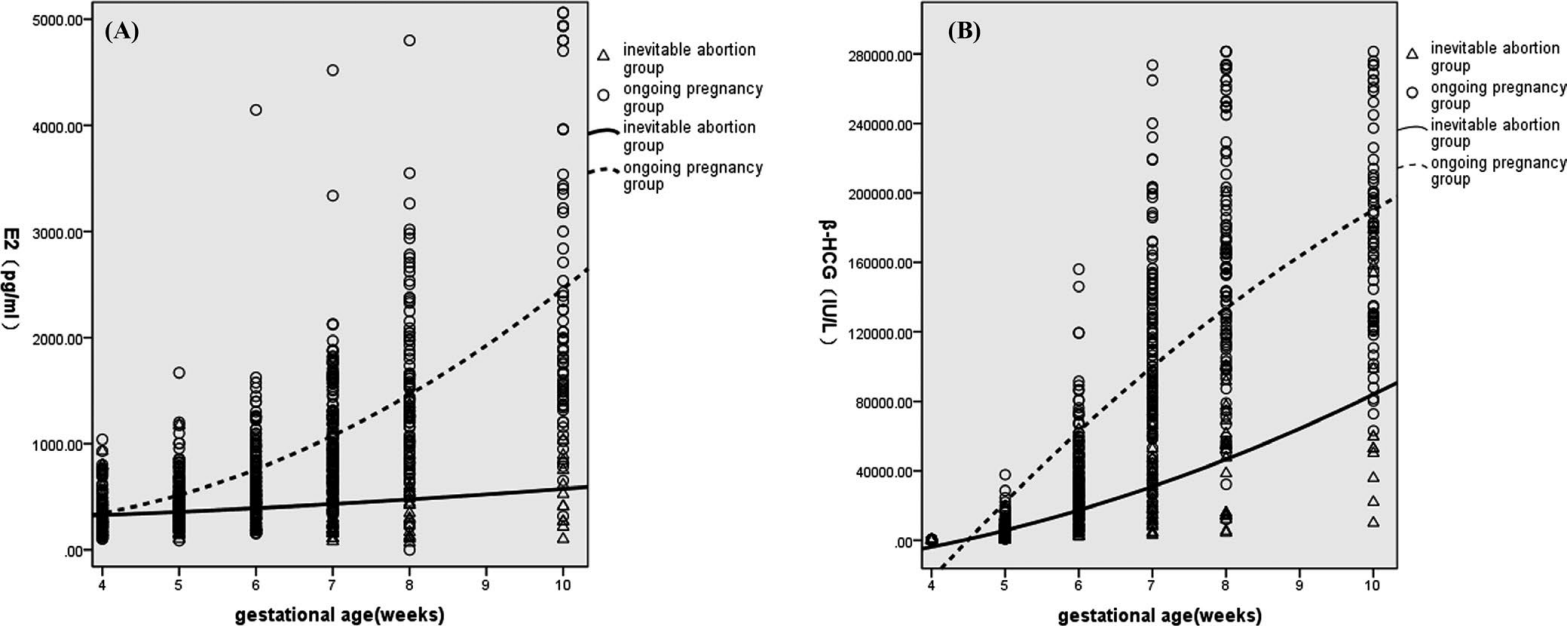
Scatter diagram and trend line (quadratic fit curve) of E2 and β‐HCG of the ongoing pregnancy group and the inevitable abortion group. A, Scatter diagram and trend line of E2 of the two groups (R2 = .436, .597); (B) Scatter diagram and trend line of β‐HCG of the two groups (R2 = .050, .475)

**Figure 2:**
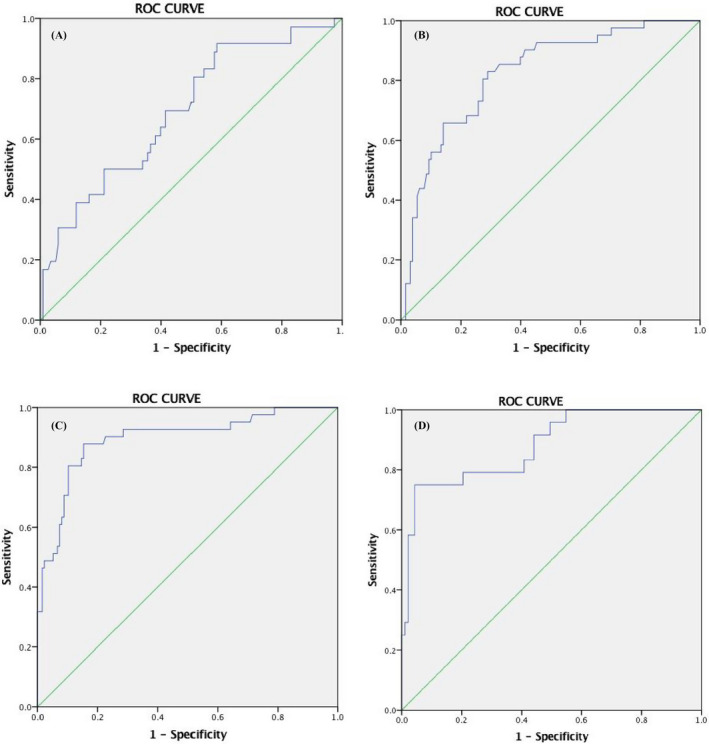
The sensitivity and specificity values of E2 level differences from 5th to 8th week of gestational age as predicted by ROC curve analyses. A: 5th week; B: 6th week; C: 7th week; and D: 8th week

We compared serum E2 and β‐HCG levels weekly between the two groups. Table 1 shows that at the 4th week of pregnancy, E2 and β‐HCG levels of the inevitable group and the ongoing pregnancy group have no remarkable differences in statistics, whereas from the 5th week to 10th week, E2 and β‐HCG levels of the ongoing pregnancy group were significantly higher than that of the inevitable abortion group. Their changes over time are depicted in Figure 1. Serum β‐HCG of the ongoing pregnancy women increased more drastically than that of the inevitable abortion women. The disparity enlarged as the gestational age increased. The E2 levels in the ongoing pregnancy group increased continuously from the 4th week to 10th week. In contrast, it showed little increase in the inevitable abortion women. Figure 2 and Table 2 depict the sensitivity and specificity values of E2 levels as predicted by ROC curve analysis from the 5th to 8th week of gestational age. We use E2 cutoff level of 489.5 pg/mL in the 5th and 6th weeks, 590.5 pg/mL in the 7th week and 614.5 pg/mL in the 8th week. The sensitivity and specificity for E2 to predict bad pregnancy outcome were 91.7% and 41.5%, 82.9% and 71.1%, 84.8% and 84.7%, and 75.0% and 95.7%, respectively (*P* < .05).

**Table 1:** Comparison of E2 and β‐HCG levels between ongoing pregnancy group and inevitable abortion group

Pregnancy week	Case number (n)		E2 (pg/mL)		β‐HCG (IU/L)	
	Ongoing pregnancy	Inevitable abortion	Ongoing pregnancy	Inevitable abortion	Ongoing pregnancy	Inevitable abortion
4th	89	26	384.25 ± 206.00	347.96 ± 217.08	201.59 ± 128.18	168.03 ± 149.32
5th	118	36	493.46 ± 249.49**	353.04 ± 201.77	6543.85 ± 6231.25**	3465.40 ± 3226.87
6th	128	41	715.67 ± 445.20**	367.78 ± 187.02	40 071.25 ± 26 393.06**	13 785.64 ± 12 308.90
7th	137	41	1077.76 ± 577.88**	411.20 ± 316.94	105 818.66 ± 51 808.84**	30 362.18 ± 22 468.49
8th	94	24	1534.39 ± 803.28**	558.53 ± 449.92	166 983.28 ± 88 657.92**	56 092.60 ± 47 904.20
10th	67	12	2424.52 ± 1346.13**	526.75 ± 299.70	171 848.34 ± 57 761.85**	77 674.71 ± 56 450.22

Note

Data are presented as mean ± standard deviation (SD) or number.

** *P* < .01.

**Table 2:** The sensitivity and specificity values of ROC curve

Pregnancy week	E2 Cutoff (pg/mL)	Specificity (%)	AUC (CI)	*P*
5th	489.5	41.5	0.69 (0.59‐0.79)	<.05
6th	489.5	71.1	0.82 (0.75‐0.90)	<.05
7th	590.5	84.7	0.90 (0.83‐0.96)	<.05
8th	614.5	95.7	0.88 (0.80‐0.96)	<.05

Growth rates of the two hormones are presented in Table 3. In the ongoing pregnancy group, the growth of E2 kept at a rate of 40%‐60% weekly. The β‐HCG level increased rapidly in the beginning of the gestational period; the growth rate slowed down as the embryo grew up. Both E2 and β‐HCG levels of the ongoing pregnancy group rose much more rapidly than those of the inevitable abortion group.

**Table 3:** Growth rates of E2 and β‐HCG in each group

Pregnancy week	E2			β‐HCG		
	Ongoing pregnancy	Inevitable abortion	*P*	Ongoing pregnancy	Inevitable abortion	*P*
4th‐5th	0.42 (0.22, 0.84)	0.13 (−0.38, 0.47)	.001	21.66 (13.34, 40.86)	17.07 (6.84, 26.47)	.038
5th‐6th	0.44 (0.17, 0.77)	0.01 (−0.21, 0.23)	.000	5.82 (3.69, 11.01)	4.04 (2.09, 7.72)	.008
6th‐7th	0.57 (0.26, 0.86)	0.04 (−1.07, 0.29)	.000	1.96 (1.30, 2.67)	1.07 (0.69, 2.18)	.000
7th‐8th	0.41 (0.17, 0.71)	−0.04 (−0.18, 0.60)	.013	0.75 (0.46, 1.09)	0.41 (0.11, 0.97)	.046

Note

Data are presented as median (quartile). Comparison were made using Mann‐Whitney *U* test.

Table 4 shows the proportion of four types of E2’s variation in each group. Eighty percent women in the ongoing pregnancy group presented continuous increase of E2 level, and no one was classified as continuous decrease or sudden drop. In the inevitable abortion group, women all failed to show continuous increase of E2, and the other three types of E2 level variations accounted for 54.0%, 34.0%, and 12.0%, respectively.

**Table 4:** Variation of E2 in the ongoing pregnancy group and the inevitable abortion group

Type of E2’s variation trend	Ongoing pregnancy group	Inevitable abortion group
Rise persistently [n (%)]	120 (80.0%)	0
Rise slowly or fluctuate [n (%)]	30 (20.0%)	27 (54.0%)
Decline continuously [n (%)]	0	17 (34.0%)
Drop suddenly [n (%)]	0	6 (12.0%)

Note

Data are presented as number (%).

## 4 DISCUSSION

The occurrence of clinical recognized pregnancy loss accounts for approximately 15%‐25% of all pregnancies.^2^ A considerable number of women suffer from recurrent pregnancy loss. In early pregnancy inspection, especially for women who present with threatened miscarriage, obstetricians and gynecologists are committed to identifying serum biological markers in order to predict pregnancy outcomes. Several studies^3^, ^4^, ^5^, ^6^ have focused on various biomarkers such as serum β‐HCG, progesterone, estradiol, PAPP‐A, inhibin, CA125, and combination of serum biomarkers and ultrasound features to predict pregnancy viability. Ideal biological markers are highly predictive, detected convenient and cost‐effective. Nowadays, the most widely used indicators are serum β‐HCG and progesterone. Progesterone is necessary in maintaining early pregnancy, playing an important role in sustaining decidualization, reducing uterine excitability, inhibiting uterine contraction, suppressing inflammatory response, and promoting maternal immune tolerance to the fetal semi‐allograft.^7^, ^8^, ^9^ However, due to large individual differences and insufficient evidence regarding appropriate progesterone cutoff levels for risk stratification of spontaneous miscarriage, its clinical value in miscarriage risk assessment remains controversial.

Human chorionic gonadotropin is a specific marker of pregnancy, synthesized in syncytiotrophoblast cells and can be detected as early as 8‐11 days following ovulation.^10^ Serum HCG level reflects quantity of trophoblast cells, rapidly increasing at the early stage of gestation, slowing down later, and reaching the peak at 8‐10 weeks of pregnancy. Previous studies^3^, ^11^ have proven quantitative determinations of HCG as a valuable tool in the clinical assessment of early pregnancy outcome. A systematic review summarized 8 studies with a total of 584 women that investigated either intact HCG or β‐HCG to predict the outcome in women with threatened miscarriage, showing a sensitivity of 44% and a specificity of 86% through further analysis using hierarchical summary receiver operating characteristic (HSROC).^6^ Liu et al reported that the optimal cutoff value of peak β‐hCG was 88 468 IU/L, with a sensitivity, specificity, positive predictive value, and negative predictive value for successful pregnancy of 95.6%, 88.0%, 95.6%, and 89.0%, respectively.^12^ Estrogen is another highly important hormone in establishing and maintaining pregnancy. Studies investigated that estrogen stimulated vascular endothelial grown factor (VEGF) production and blood vessel formation to enable maternal‐fetal circulation,^13^ and showed functions in immune regulation.^14^ It modulates the immune response by inducing peripheral T cells to secrete the pro‐inflammatory cytokines.^14^ Xu et al^15^ compared sex hormone and sex hormone metabolite levels of women in early pregnancy with and without threatened miscarriage, and reported that serum E2 levels were lower in women with threatened miscarriage. They proposed that abnormal levels of sex hormone metabolites and reduction of estrogen activity might result in bleeding during the first trimester of pregnancy. Compared to progesterone and HCG, estrogen is not as widely used in pregnancy assessment. Previous study has found that the sensitivities of estradiol (80%) and HCG (85%) in predicting pregnancy outcome at week 8 of gestation were better than that of serum progesterone (56%).^16^ Pillai's review found similar results, but the sensitivity was heterogeneous among previous reported studies.^6^

As the levels of serum β‐HCG and estradiol change over each week of gestation, most of the previous studies did not take this into consideration. A few reports have examined changes of E2 in early pregnancy. The present study focused on dynamic variation of E2 and β‐HCG in early pregnancy women, examining their levels and growth rates to help us predict the miscarriage risk accurately and timely. The results presented that β‐HCG and E2 levels of the ongoing pregnancy group were significantly higher and increased more rapidly than those of the inevitable abortion group. β‐HCG in normal pregnancy increased drastically within the beginning period of gestation, and from the 5th week to 8th week, medians of the growth rate were 5.82, 1.96, and 0.75 per week. Low growth rate may predict bad pregnancy outcome. Quantitative value and growth rate are both meaningful in early pregnancy assessment. Although low β‐HCG level and slow increase rate probably present bad outcome for most women, some patients conceive well‐developed fetus and get good pregnancy outcome who present unsatisfactory β‐HCG values and growth rates in clinical practice. When β‐HCG combined with E2, the prediction of pregnancy outcome will be more accurate. Miscarriage women showed lower E2 levels. E2 in normal pregnancy women increased 40%‐60% weekly on average. Slow growth rate and decreased E2 level are likely to predict bad outcome. Results of our study agreed with most other studies; moreover, we provided more information about the variations and growth rates of women with different early pregnancy outcomes.

The major limitation of our study is the relatively small sample size, especially in the inevitable abortion group. It is a little difficult to get large amount of data of miscarriage women due to a relatively small number of miscarriage patients and lack of hormone values after their abortions. So in the future study, we will make more efforts to collect larger samples to strengthen the results and hope to find more highly predictive and cost‐effective biological markers in predicting pregnancy outcome.

In conclusion, dynamic monitoring of maternal serum E2 and β‐HCG levels has important clinical implication for early pregnancy assessment and pregnancy outcome prediction. Low values and low growth rates of E2 and β‐HCG probably indicate bad pregnancy outcomes.
